# Near-Infrared Spectroscopy for Rapid Determination of Physicochemical Properties of Fermented Fish Sauce (‘Nam Pla-Ra’)

**DOI:** 10.17113/ftb.64.02.26.8998

**Published:** 2026-06-15

**Authors:** Sunee Jungtheerapanich, Kraireuk Ngowsuwan, Sumaporn Kasemsumran

**Affiliations:** Laboratory of Non-Destructive Quality Evaluation of Commodities, Kasetsart Agricultural and Agro-Industrial Product Improvement Institute (KAPI), Kasetsart University, 50 Ngam Wong Wan Road, Bangkok 10900, Thailand

**Keywords:** fermented fish sauce (nam pla-ra), near infrared spectroscopy, determination of physicochemical properties, predictive model

## Abstract

**Research background:**

Thai community product quality standards have been established for nam pla-ra, which require at least 4 % protein and 12 % sodium chloride. Determining the protein and NaCl contents requires chemicals and is time-consuming. In addition to reporting protein and salt content, other quality controls must also be carried out. Thus, the objective of this research is to develop predictive models for the determination of physicochemical properties of nam pla-ra using near-infrared spectroscopy (NIRS) for fast and simultaneous multi-parameter prediction.

**Experimental approach:**

The physicochemical values of commercially available fermented fish sauce (‘nam pla-ra’ in Thai) were evaluated to develop predictive models for determining physicochemical values and developing models to categorize samples. The model was developed and tested based on the seven parameters: total soluble solids (TSS), pH, L*, a*, b*, NaCl and protein content.

**Results and conclusions:**

The predictive models for pH and colour (L*, a*, b*) could be used for screening, based on their coefficients of determination (R^2^=0.73–0.81; SEP=0.15–0.94). The model for determining protein content (R^2^=0.86; SEP=0.44) is suitable for screening, routine quality control, and research applications. In contrast, the TSS and NaCl models (R^2^=0.97–0.98; SEP=0.41–0.61) demonstrate high predictive performance and can be reliably applied to all analytical tasks, including screening, routine quality control, and research purposes. The physicochemical properties of nam pla-ra could be predicted accurately using TSS, colour (L*, a*, b*), NaCl, and protein content. There was no significant (p>0.05) difference between the quality values obtained from the predictive model and the reference method, except for the pH value.

**Novelty and scientific contribution:**

NIRS showed potential in predicting the physicochemical properties of nam pla-ra using TSS and colour (L*, a*, b*) in the range 1000–2500 nm, and NaCl and protein contents in the selected ranges 1000–1889 and 2031–2408 nm, respectively. Furthermore, these findings demonstrate the potential of NIRS as a rapid, non-destructive, environmentally friendly and reliable analytical tool for determining the properties of nam pla-ra. NIRS can contribute to ensuring product consistency and facilitating standardisation in the fermented fish sauce industry. Such applications enhance food safety and consumer confidence.

## INTRODUCTION

Fermented fish (‘pla-ra’ in Thai) is produced from freshwater fish, such as *Channa striata* (‘chon’ in Thai), *Trichogaster trichopterus* (‘kra-dee’), *Cyclocheilichthys repasson* (‘soi’) and *Puntius gonionotus* (‘tapien’), which are fermented with salt and rice bran or roasted rice powder in a closed container for 6 to 12 months at ambient temperature. This method is important for the preservation of high-protein foods (*1*, *2*).

A product called fermented fish sauce (‘nam pla-ra’ in Thai) is made from pla-ra and is seasoned with ingredients including sugar, tamarind juice and pickled garlic juice. It is then filtered, cooked, and packaged (*3*). In Thailand, particularly in the north and northeast, nam pla-ra is primarily used as an ingredient in regional dishes, such as curries, soups and spicy salads.

Initially, small- and medium-scale manufacturing of pla-ra and nam pla-ra focused on domestic or local consumption. Pla-ra and nam pla-ra are now in great demand due to migration and the sharing of food cultures. Pla-ra production in Thailand is approx. 40 000 tonnes per year, worth almost THB 800 million (approx. EUR 20 million) per year, while the value of pla-ra for export (to Laos, Vietnam, Cambodia, the United States, the European Union, and Middle Eastern nations) is more than THB 20 million per year (*4*). Consequently, Thai community product quality standards have been established for nam pla-ra, requiring it to contain at least 4 % protein and 12 % salt (*3*).

Determining the protein and sodium chloride contents requires the use of chemicals and is time-consuming, while near-infrared spectroscopy (NIRS) is a powerful tool for determining physicochemical values that is rapid, practical, environmentally safe, and requires little sample preparation. This technique can be used for both quantitative and qualitative studies of food products and it can determine several physicochemical values in a single scan (*5*–*8*). Other studies have successfully evaluated the quality of alcoholic and acid fermentations from low-grade fruits using NIRS (*9*, *10*). However, other fermented products with nontransparent properties, such as nam pla-ra sauce, are very challenging to investigate using the NIRS method. In fish or other sauces, NIRS can be used to determine physicochemical parameters such as total soluble solid (TSS), total nitrogen content, and pH of fish sauce (*11*), final quality of soy sauce (shoyu) (*12*), total nitrogen content in soy sauce (*13*), and sensory quality of Japanese fermented soya bean paste (miso) (*14*). However, no published studies have mentioned the use of NIRS for predicting physicochemical properties of nam pla-ra, such as the protein and NaCl contents, which are important parameters to be controlled. In addition to standard efforts, further quality control parameters of TSS, pH and colour (L*, a*, b*) are also included in this study. Thus, the objective of this research is to develop predictive models for determining nam pla-ra physicochemical properties using NIRS for fast and simultaneous multi-parameter prediction.

## MATERIALS AND METHODS

### Material

Three brands of nam pla-ra, each with a different sodium chloride and protein content (low, medium and high), were chosen from regional and local supermarkets in Bangkok and Pathum Thani Province, Thailand. They were used without further treatment and mixed using the mixture design technique, resulting in a total of 60 samples (Table S1). An additional forty brands from the market were obtained. All samples (*N*=100) were used to develop the calibration and prediction models for the physicochemical values of nam pla-ra. Information on nam pla-ra manufacturers, is given in Table S2.

### FT-NIR analysis

A volume of 30 mL of nam pla-ra from each sample was placed in separate plastic tubes and incubated in a water bath at 30 °C for 30 min. Each sample was then placed in a glass Petri dish with a transflectance cover and analysed using a Fourier transform near-infrared (FT-NIR) spectrophotometer (model NIR Flex N-500; Buchi, Flawil, Switzerland) in transflectance mode. All spectra were collected in the wavenumber range 10 000–4000 cm^-1^ (1000–2500 nm) with 64 times per scan and a resolution of 8 cm^-1^. A single spectrum was produced for each sample by averaging the three acquired spectra.

### Physicochemical characterisation of nam pla-ra by reference methods

The physicochemical properties of nam pla-ra, namely total soluble solids (TSS), pH, colour (L*, a*, b*), sodium chloride and protein content, were determined using standard methods. Three measurements were carried out per sample.

TSS were measured using a pocket refractometer (model PAL-1; Atago, Tokyo, Japan). The pH value was determined at 25 °C using a pH meter (model CG842; Schott, Mainz, Germany). The colour (L*, a*, b*) of each sample was measured using a spectrophotometer (model CM-700d; Konica Minolta, Osaka, Japan).

To determine the NaCl content, a sample (1 g) was weighed into a beaker with 200 mL of deionized water. The mixture was adjusted to neutral pH with 0.1 M sodium hydrogen carbonate solution (Elago Enterprises Pty Ltd, Castle Hill, NSW, Australia) and the volume was brought to 250 mL with deionized water. A sample (10 mL) was pipetted into an Erlenmeyer flask, and 1 mL of 5 % potassium chromate solution (Elago Enterprises Pty Ltd) was added. The sample was titrated with 0.01 M silver nitrate solution (Avantor Performance Materials Poland S.A., Gliwice, Poland) until the end point (brick red sediment). The NaCl content was calculated using the following equation (*15*):


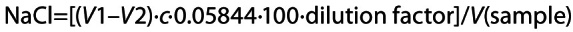
 /1/

where *V*1 and *V*2 are the volumes of silver nitrate solution titrated with the sample (mL) and the blank (mL), respectively, *c* is the concentration of silver nitrate solution (M), and 0.05844 is the molar mass of NaCl expressed in g/mmol, which is used as a conversion factor to calculate the NaCl content.

The protein content was determined according to the AOAC method (*16*) using the Kjeldahl method. A sample (2 g) was weighed into a protein digestion tube. Then, 10 g of selenium mixture and 15 mL of sulfuric acid were added. A blank was prepared in parallel using distilled water instead of the sample. The samples were digested and allowed to cool to room temperature. After digestion, the samples were distilled by adding 25 mL of 4 % boric acid solution into a 250-mL flask containing 2 drops of indicator solution, which was used to collect the distillate. The distillate was then titrated with 0.1 M hydrochloric acid solution until the end point was reached. The volume of 0.1 M hydrochloric acid solution consumed was recorded. The protein content was calculated using the following equations:


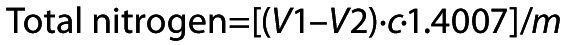
 /2/


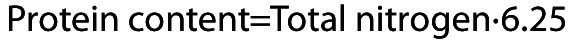
 /3/

where *V*1 and *V*2 are the volumes of hydrochloric acid solution used in the titration of the sample (mL) and the blank (mL), respectively, *c* is the concentration of hydrochloric acid solution (M), and *m* is the sample mass (g).

### NIR spectroscopic model development

#### Predictive model development

In the development of predictive models, the standard sequence involves calibration, validation and prediction. For this pilot study, however, the validation procedure was implemented instead of the prediction stage. The measurement data and NIR spectra (full and selected spectra) were separated into two groups: the calibration set (*N*=75) and the prediction set (*N*=25). Unscrambler software v. 9.8 (*17*) was used to create chemometric models with partial least square (PLS) regression with both original and pretreated spectra. Spectral preprocessing included second derivatives based on the Savitzky-Golay method (polynomial order=2) and standard normal variate (SNV) to reduce signal variation caused by light scattering effects in the samples and to enhance the predictive accuracy and stability of the model. The coefficient of determination (R^2^), standard error of calibration (SEC), standard error of prediction (SEP), and bias were used to describe the performance of each model. The optimal model was selected based on the lowest SEC, SEP, and bias, and the highest R^2^.

#### Validation of the predictive model

The predictive sample set (*N*=25) was used to validate the predictive model in the Unscrambler software (*17*). Subsequently, statistical tests were conducted using a paired sample *t*-test, with significance assessed at the p<0.05 level between the measured value from the reference method and the predicted model, using IBM SPSS Statistics v. 17.0 software (*18*). The significance (2-tailed) and bias were used to describe the performance of the model.

## RESULTS AND DISCUSSION

### Physicochemical values of nam pla-ra by reference method

Table 1 shows the properties of nam pla-ra as determined by the reference technique, including total soluble solids (TSS), pH, colour (L*, a*, b*), sodium chloride and protein content. The physicochemical properties (TSS, pH, colour, NaCl and protein content) and the spectral results were divided into two groups to develop the predictive model, with a calibration set (*N*=75) and a prediction set (*N*=25). The ranges of all physicochemical reference values in the calibration set fully encompassed those observed in the prediction set.

**Table 1:** Composition variation and properties of nam pla-ra samples used to develop partial least square (PLS) models

Parameter	Calibration set (*N*=75)				Prediction set (*N*=25)			
	Min	Max	Mean	S.D.	Min	Max	Mean	S.D.
*w*(TSS)/%	13.93	48.50	28.35	4.80	22.77	41.10	28.76	4.45
pH	4.08	6.06	5.03	0.39	4.42	5.90	5.06	0.39
L*	25.97	36.51	32.13	2.28	28.16	36.48	32.36	2.21
a*	1.31	7.59	3.47	0.79	2.41	6.20	3.52	0.73
b*	1.69	10.82	6.88	1.77	3.99	9.83	7.03	1.65
*w*(NaCl)/%	8.38	21.87	15.80	2.62	11.35	21.57	16.08	2.49
*w*(protein)/%	1.57	7.82	3.50	1.13	2.06	6.94	3.59	1.16

TSS=total soluble solids

### Results of FT-NIR analysis

**Fig. 1:**
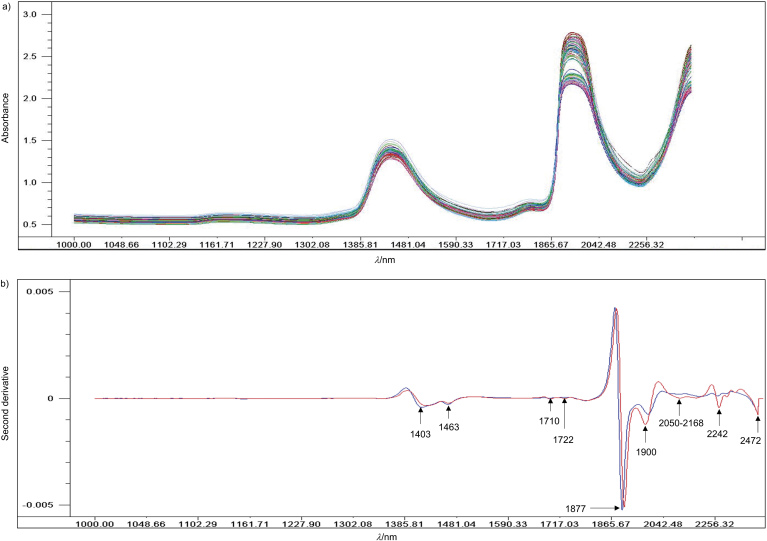
NIR spectra of: a) Original nam pla-ra product, and b) after the pretreatment with second derivative Savitzky-Golay method

The NIR spectra of nam pla-ra samples were obtained using an FT-NIR spectrophotometer in transflectance mode and the spectral profiles are shown in Fig. 1a.

Fig. 1b shows the NIR spectra of samples with the highest and lowest protein content, recorded on FT-NIR and pretreated with the second derivative Savitzky-Golay method for important band assignment, while Table 2 (*19*-*24*) shows the band assignment data. Water is a key component in nam pla-ra. At 1403, 1463 and 1877–1900 nm, it exhibited strong absorption bands resulting from the combination of O-H overtone, stretching, and bending wavelengths (*19*). The bands associated with pH (lactic acid from the fermentation and the ionization of lactate from lactic acid) were observed at 1710, 1722, 1730, 1750, 1890, 2160 and 2470 nm as the first overtones of C-H stretching, O-H stretching combined with C=O stretching, C-H stretching, C=O stretching combination, and C-H stretching combined with C=O stretching (*20*–*24*). The absorption bands for protein in the spectral region 2050–2168 nm resulted from the N-H stretching combination and the N-H band second overtone (*24*). At 2242 and 2472 nm, combinations of C-H stretching, CH_2_ deformation, C-C stretching combination, and C-O-C stretching combination were related to sugar (*24*). Furthermore, although NaCl lacks unique NIR absorption bands, the effect of salt on the water absorption band enabled the detection of changes in salt content. The strength of the water band absorption decreased as the salt content increased (*19*, *25*). For TSS, this can be attributed to NaCl, as the main soluble solid dissolved in nam pla-ra. NIRS does not directly measure the L*, a*, b* colour parameters of fermented fish sauce as human vision or a colorimeter does. Instead, it measures light absorption or reflection in the near-infrared range, which is indirectly correlated with the chemical composition and physical structure affecting the colour of the product through precise chemometric modelling. In nam pla-ra, the brown to dark brown colour is due to compounds containing C-H, O-H, and N-H bonds, which are detectable by NIRS. Variations in these colour-forming compounds alter the NIR spectrum. Specifically, their concentrations, which determine L*, a* and b* colour values, correlate with light absorption in the NIR range.

**Table 2:** Band assignments of significant near-infra red (NIR) regions of nam pla-ra

Wavelength/nm	Band assignment	Substance
1403	O-H first overtone	Water (*19*)
1463	O-H first overtone	Water (*19*)
1710–1750	first overtones of the C-H stretching	Lactic acid, lactate (*20*–*24*)
1877–1900	O-H stretching, bending combination band	Water (*19*)
1890	O-H stretching combined with C=O stretching	Carboxylic acid (*24*)
2050–2168	N-H stretching combination, N-H band second overtone	Protein (*24*)
2160	C-H stretching, C=O stretching combination	Carboxylic acid (*24*)
2242	C-H stretching, CH_2_ deformation combination	Starch, sugar (*24*)
2470	C-H stretching combined with C=O stretching	Carboxylic acid (*24*)
2472	C-H stretching, C-C stretching combination, C-O-C stretching combination	Starch, sugar (*24*)

**Fig. 2:**
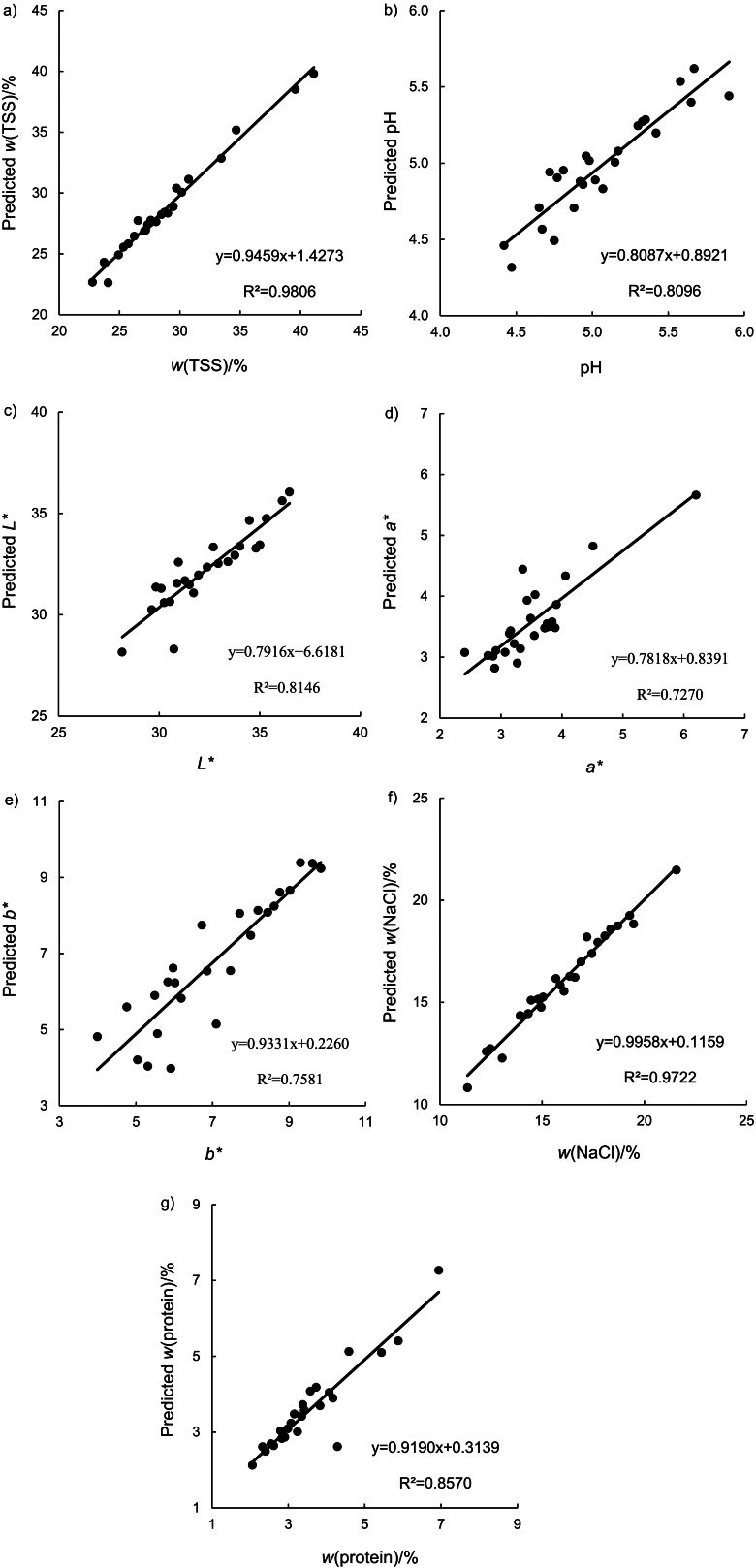
Scatter plots for comparison of measured and predicted values (from full spectrum) for nam pla-ra qualities of prediction set for: a) TSS, b) pH, c) L*, d) a*, e) b*, f) sodium chloride content, and g) protein content. TSS=total soluble solids

**Fig. 3:**
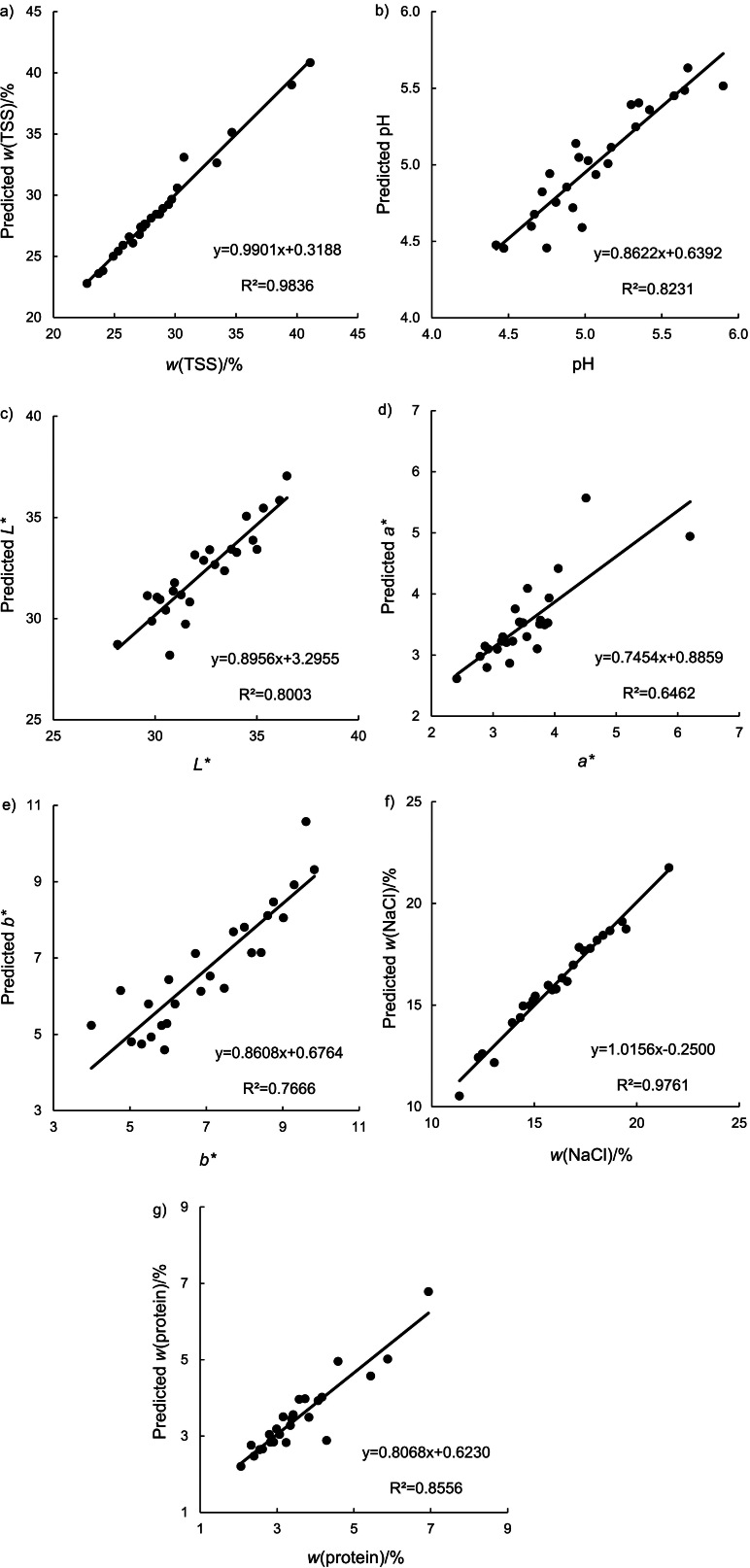
Scatter plots for comparison of measured and predicted values (from selected spectrum) for nam pla-ra qualities of prediction set for: a) TSS, b) pH, c) L*, d) a*, e) b*, f) sodium chloride content, and g) protein content. TSS=total soluble solids

Calibration models for physicochemical values were developed using PLS regression. The calibration and prediction statistics are shown in Table 3, and scatter plots comparing the measured and predicted values for each parameter are shown in Fig. 2 (models using the full spectrum in the range 1000–2500 nm) and Fig. 3 (models using the selected spectrum in the ranges 1000–1889 and 2031–2408 nm to avoid overlapping and over-absorption bands (*26*)). The performance of the PLS calibration models was evaluated using the coefficient of determination (R^2^), the standard error of calibration (SEC), and the standard error of prediction (SEP). As shown in Table 3, the number of factors indicates the optimal number of latent variables used in the PLS regression models, selected to balance model accuracy and complexity while avoiding overfitting. In this study, the optimal number of factors ranged from 4 to 22, depending on the physicochemical parameter and the spectral preprocessing method applied. The use of an appropriate number of factors helped minimise prediction errors, indicating good model stability and generalisation capability. Overall, the calibration models showed relatively high R^2^ values, demonstrating strong agreement between the measured and predicted physicochemical properties. In addition, the SEC and SEP values were generally low and comparable for most parameters, suggesting satisfactory calibration accuracy and reliable prediction performance. For the full spectrum model, the original spectrum produced the best results for pH, colour (L* and a*) and NaCl. The second derivative of NIR spectra using the Savitzky-Golay method was optimal for colour (b*), while the standard normal variate (SNV) method was optimal for TSS and protein content. For the selected spectrum model, the original spectrum was optimal for TSS, colour (b*) and NaCl. The second derivative of NIR spectra using the Savitzky-Golay method was optimal for pH and protein content, while the SNV method was optimal for colour (L* and a*).

**Table 3:** Partial least square (PLS) model statistics for nam pla-ra physicochemical values

Parameter	Pretreatment	Factor	Calibration (*N*=75)			Prediction (*N*=25)		
			R^2^	SEC	Bias	R^2^	SEP	Bias
Full spectrum range (1000–2500 nm)								
*w*(TSS)/%	None	5	0.9716	0.8093	1.767⋅10^-6^	0.9756	0.6762	-0.1574
	2D (11,11,2)	5	0.9839	0.6086	9.664⋅10^7^	0.9748	0.7056	-0.0163
	SNV	4	0.9732	0.7856	9.664⋅10^7^	0.9806	0.6062	-0.1276
pH	None	22	0.9852	0.0470	-9.549⋅10^-6^	0.8096	0.1496	-0.0762
	2D (11,11,2)	13	0.9430	0.0922	-2.352⋅10^-7^	0.7677	0.1705	-0.0733
	SNV	19	0.9755	0.0605	-3.236⋅10^-6^	0.7866	0.1654	-0.0655
L*	None	8	0.8913	0.7518	4.578⋅10^-7^	0.8146	0.9434	-0.1265
	2D (7,7,2)	4	0.7581	1.1215	5.086⋅10^-7^	0.7844	1.0266	-0.0240
	SNV	7	0.8845	0.7751	2.797⋅10^-7^	0.7951	1.0000	0.0445
a*	None	9	0.8336	0.3239	8.074⋅10^-7^	0.7270	0.3725	0.0699
	2D (3,3,2)	4	0.7236	0.4175	6.358⋅10^-9^	0.3423	0.5885	0.0078
	SNV	13	0.9294	0.2110	4.768⋅10^-7^	0.6200	0.4457	-0.0386
b*	None	9	0.8672	0.6435	-1.367⋅10^-6^	0.7554	0.8138	-0.0754
	2D (11,11,2)	8	0.8135	0.7626	-1.717⋅10^-7^	0.7581	0.7739	-0.2439
	SNV	6	0.8268	0.7349	9.219⋅10^-8^	0.7570	0.8147	0.0005
*w*(NaCl)/%	None	6	0.9822	0.3494	-1.030⋅10^-6^	0.9722	0.4116	0.0491
	2D (11,11,2)	5	0.9668	0.4763	3.815⋅10^-8^	0.7793	1.1448	-0.2284
	SNV	3	0.9728	0.4315	-6.485⋅10^-7^	0.9685	0.4410	-0.0111
*w*(protein)/%	None	7	0.8996	0.3576	-2.480⋅10^-7^	0.8544	0.4414	0.0091
	2D (11,11,2)	12	0.9623	0.2190	4.292⋅10^-8^	0.8292	0.4781	0.0065
	SNV	5	0.8903	0.3737	-2.750⋅10^-7^	0.8570	0.4368	0.0233
Selected spectrum range (1000–1889 and 2031–2408 nm)								
*w*(TSS)/%	None	6	0.9885	0.5151	1.284⋅10^-6^	0.9836	0.5677	0.0339
	2D (11,11,2)	4	0.9840	0.6069	6.358⋅10^-7^	0.9649	0.8267	0.0975
	SNV	4	0.9784	0.7064	8.901⋅10^-7^	0.9682	0.7901	0.0697
pH	None	13	0.8965	0.1243	3.815⋅10^-7^	0.6240	0.2337	-0.0378
	2D (11,11,2)	12	0.9421	0.0930	-9.537⋅10^-8^	0.8231	0.1511	-0.0584
	SNV	14	0.9084	0.1169	-6.104⋅10^-7^	0.6697	0.2191	-0.0350
L*	None	4	0.7526	1.1343	1.780⋅10^-7^	0.6998	1.2075	-0.0993
	2D (7,7,2)	7	0.8462	0.8942	2.391⋅10^-6^	0.7949	0.9956	0.1076
	SNV	13	0.9498	0.5110	-3.942⋅10^-6^	0.8003	0.9846	-0.0829
a*	None	12	0.9042	0.2458	1.295⋅10^-6^	0.6166	0.4481	0.0340
	2D (9,9,2)	7	0.7880	0.3656	-1.653⋅10^-7^	0.4320	0.5444	-0.0522
	SNV	11	0.9002	0.2509	4.689⋅10^-7^	0.6462	0.4316	-0.0113
b*	None	8	0.8322	0.7234	-6.994⋅10^-7^	0.7666	0.7367	-0.3018
	2D (9,9,2)	7	0.8246	0.7395	-5.563⋅10^-7^	0.6949	0.8875	-0.2096
	SNV	8	0.8385	0.7097	-6.358⋅10^-8^	0.7117	0.8852	-0.0609
*w*(NaCl)/%	None	5	0.9845	0.3252	-5.468⋅10^-7^	0.9761	0.3843	0.0009
	2D (9,9,2)	5	0.9796	0.3730	-2.925⋅10^-7^	0.9164	0.7186	-0.0308
	SNV	4	0.9904	0.2566	-2.797⋅10^-7^	0.9718	0.4175	-0.0151
*w*(protein)/%	None	9	0.9342	0.2893	-5.817⋅10^-7^	0.8109	0.4961	-0.0812
	2D (11,11,2)	7	0.9336	0.2907	-1.208⋅10^-7^	0.8556	0.4338	-0.0695
	SNV	7	0.9256	0.3078	8.424⋅10^-8^	0.8347	0.4676	-0.0500

The best model for each parameter is shown in orange. Spectral regions for model development (selected spectrum) were 1000–1889 and 2031–2408 nm. Pretreatments: 2D=second derivatives, SNV=standard normal variate. PLS model statistics: R^2^=coefficient of determination, SEC=standard error of calibration, SEP=standard error of prediction

The comparison between the full spectrum and selected spectrum models showed that the best predictive model from the full spectrum included pH and colour (L* and a*). In contrast, the best predictive model from the selected spectrum was developed using TSS, colour (b*), NaCl, and protein content. The full spectrum provided the best model for physical properties, since colour may arise from physical properties that do not have distinct absorption bands in the NIR. The best model for physical parameters was produced using the full spectrum (representative of all functions) predictive model, while the selected spectrum gave the best model for chemical properties (TSS, NaCl and protein content), possibly because these properties corresponded to specific absorption bands. Thus, the selected spectrum (without the broad band for water) provided the best model for specific absorption bands. Furthermore, the predictive models for pH and colour (L*, a*, b*) may be used for screening (R^2^=0.73–0.81); the protein content models can be used for research and general purposes (R^2^=0.86); and the TSS and NaCl models can be used for all types of work (R^2^=0.97–0.98) (*27*).

Compared with previous studies, Ritthiruangdej *et al*. (*11*) applied NIRS in the spectral range of 1100–2500 nm to predict total nitrogen (TN), pH, refractive index, density and Brix in Thai fish sauces. Their results showed that the informative spectral regions selected by searching combination moving window partial least squares (SCMWPLS) were strongly associated with specific chemical properties, including the regions of 2264–2428 nm for TN, 1698–1722 and 2222–2258 nm for pH, 1358–1438 nm for density, 1774–1846 and 2078–2114 nm for refractive index, and 1322–1442 and 2000–2076 nm for Brix. In addition, qualitative classification models based on TN content achieved correct classification rates exceeding 82 %. These findings are consistent with the results of the present study, demonstrating that variable selection and the use of informative spectral regions can improve the prediction of chemical attributes related to distinct NIR absorption bands, while full spectrum models are more suitable for predicting physical properties. Similarly, Jiang *et al*. (*28*) investigated the application of dual-band NIRS for the non-destructive determination of fat, protein, collagen, ash and sodium contents in meat stewed in soy sauce. Using spectral ranges of 650–950 and 960–1660 nm, PLS models were developed based on spectra collected from vacuum-packed ready-to-eat products from 97 different brands. The results demonstrated that fat and protein contents were predicted with the highest accuracy, with superior model performance obtained in the 960–1660 nm region compared to the 650–950 nm range. These findings are in agreement with the present study, emphasising that the selection of appropriate spectral regions enhances predictive performance for chemical constituents associated with specific absorption bands. Furthermore, Zhang *et al*. (*29*) collected NIR spectra in the range of 1000–2500 nm using a miniature fibre-optic NIR spectrometer to monitor different stages of soy sauce production, including steamed soybean, koji and moromi. Their study successfully developed predictive models for *in situ* and real-time assessment of the digestion rate of steamed soybean, protease activity in koji, and formaldehyde nitrogen content in moromi. These results further support the suitability of NIRS for predicting key chemical attributes in fermented food systems, which is consistent with the findings of the present study. Nevertheless, there have been no reported studies on the quantification of physicochemical properties of nam pla-ra using NIRS.

### Predictive model validation

The predictive model validation showed that the developed model accurately predicted nam pla-ra physicochemical properties (TSS, colour (L*, a*, b*), NaCl and protein content). There was no significant difference between the physicochemical values obtained from the predictive model and those from the reference method, except for the pH value (Table 4) possibly because the pH value in the samples had a relatively narrow range. The pH value (Table 1) had the lowest standard deviation value (0.38) compared to other physicochemical values. Therefore, the accuracy of the pH value may still be affected by the prediction model, even if it has a low bias value. Consequently, the outputs of the prediction model and the reference method were different.

**Table 4:** Statistics of predictive model validation for nam pla-ra physicochemical values

Parameter	Number of samples	Selected model	Bias	Significance (2-tailed)
*w*(TSS)/%	25	Selected spectrum (non-pretreatment)	0.0339	0.768*
pH	25	Full spectrum (non-pretreatment)	-0.0762	0.018
L*	25	Full spectrum (non-pretreatment)	-0.1265	0.509*
a*	25	Full spectrum (non-pretreatment)	0.0699	0.358*
b*	25	Selected spectrum (non- pretreatment)	-0.3018	0.052*
*w*(NaCl)/%	25	Selected spectrum (non- pretreatment)	0.0009	0.990*
*w*(protein)/%	25	Selected spectrum (2D (11,11,2) pretreatment)	-0.0695	0.432*

*Indicates no significant difference between the quality value from the reference method and the predictive model at α=0.05

## CONCLUSIONS

In summary, the physicochemical properties of nam pla-ra included total soluble solid (TSS) (13.93–48.50 %), pH (4.08–6.06), L* (25.97–36.51), a* (1.31–7.59), b* (1.69–10.82), sodium chloride content (8.38–21.87 %) and protein content (1.57–7.82 %). Partial least squares (PLS) regressions were developed between the FT-NIR spectra (full and selected) and the physicochemical properties of nam pla-ra. These included TSS, pH, colour (L*, a*, b*), NaCl and protein content. The findings clearly indicate that for predicting physical properties or parameters involving complex overall composition, such as pH, L* or a* values, the most accurate models are those developed using full-spectrum data. In contrast, for predicting specific chemical parameters such as TSS, NaCl and protein content, as well as the b* value (yellow-blue axis), which relates to the chemical components responsible for the characteristic colour of fermented fish meat, the optimal models are derived from selected spectral regions. This supports the immediate application of selecting wavelength ranges that correlate with physicochemical properties. In addition, it was found that pH and colour (L*, a*, b*) models could be used for screening, the protein content model for screening, routine quality control, and research applications, and the TSS and NaCl models for all analytical tasks, including screening, routine quality control, and research purposes. The developed model could accurately predict nam pla-ra physicochemical values (TSS, colour (L*, a*, b*), NaCl and protein content). There was no significant difference (p>0.05) in physicochemical properties between the predictive model and the reference method, except for the pH value. Finally, the NIR technique outperforms conventional methods in determining multiple quality parameters simultaneously (TSS, colour, protein, and NaCl) for nam pla-ra, requiring less than three minutes per scan.

## SUPPLEMENTARY MATERIALS

Supplementary materials are available at: https://www.ftb.com.hr/images/pdfarticles/2026/April-June/FTB-64-140-S1.pdf.

## Appendix

**Table S1:** Details about the mixture design technique

Mixture design technique	Sample	*w*(sample)		
		A	B	C
Simplex lattice design	1	1	0	0
	2	0	0	1
	3	0	1	0
	4	0.67	0	0.33
	5	0.67	0.33	0
	6	0.33	0	0.67
	7	0.33	0.67	0
	8	0	0.33	0.67
	9	0	0.67	0.33
	10	0.33	0.33	0.33
	11	1	0	0
	12	0	0	1
	13	0	1	0
	14	0.67	0	0.33
	15	0.67	0.33	0
	16	0.33	0	0.67
	17	0.33	0.67	0
	18	0	0.33	0.67
	19	0	0.67	0.33
	20	0.33	0.33	0.33
	21	1	0	0
	22	0	0	1
	23	0	1	0
	24	0.67	0	0.33
	25	0.67	0.33	0
	26	0.33	0	0.67
	27	0.33	0.67	0
	28	0	0.33	0.67
	29	0	0.67	0.33
	30	0.33	0.33	0.33
Simplex axial design	31	1	0	0
	32	0	0	1
	33	0	1	0
	34	0.5	0.5	0
	35	0.5	0	0.5
	36	0	0.5	0.5
	37	0.67	0.16	0.16
	38	0.16	0.16	0.67
	39	0.16	0.67	0.16
	40	0.33	0.33	0.33
	41	1	0	0
	42	0	0	1
	43	0	1	0
	44	0.5	0.5	0
	45	0.5	0	0.5
	46	0	0.5	0.5
	47	0.67	0.16	0.16
	48	0.16	0.16	0.67
	49	0.16	0.67	0.16
	50	0.33	0.33	0.33
	51	1	0	0
	52	0	0	1
	53	0	1	0
	54	0.5	0.5	0
	55	0.5	0	0.5
	56	0	0.5	0.5
	57	0.67	0.16	0.16
	58	0.16	0.16	0.67
	59	0.16	0.67	0.16
	60	0.33	0.33	0.33

A=low sodium chloride and protein content, B=medium sodium chloride and protein content, C=high sodium chloride and protein content

**Table S2:** Information about nam pla-ra (manufacturer, city, province, and country of origin)

Manufacturer	Origin (city, province, country)
Zen and Kosum Interfoods Co., Ltd.	Kosum Phisai, Maha Sarakham, Thailand
Piromporn Agriculture Co., Ltd.	Lat Phrao, Bangkok, Thailand
Setthisaeb Limited Partnership	Thawat Buri, Roi Et, Thailand
Thai Kori Nomimono Co., Ltd.	Mueang Sakon Nakhon, Sakon Nakhon, Thailand
Pickled Fish Factory Mother Prakas Co., Ltd.	Sam Sung, Khon Kaen, Thailand
Mekhala Inter Foods Co., Ltd.	Mueang Samut Prakan, Samut Prakan, Thailand
Jawirat Food Co., Ltd	Phayuha Khiri, Nakhon Sawan, Thailand
Native Food Co., Ltd	Wihan Daeng, Saraburi, Thailand
Plara Racha Co., Ltd	Lat Lum Kaeo, Pathum Thani, Thailand
Phetdam Foods Co., Ltd.	Mueang Kalasin, Kalasin, Thailand
Nongporn Food Industries Co., Ltd.	Bang Bua Thong, Nonthaburi, Thailand
Plara Maerien Co., Ltd.	Erawan, Loei, Thailand
Sudjai Jarernkankha Co., Ltd.	Khanu Woralaksaburi, Kamphaeng Phet, Thailand
Yan Wal Yun Co., Ltd.	Mueang Samut Sakhon, Samut Sakhon, **Thailand**
Siriporn (1622) Co., Ltd	Thawat Buri, Roi Et, Thailand
K.S.F Foods Products Co., Ltd	Kosum Phisai, Maha Sarakham, Thailand
Elah Kumpang Interfoods Co., Ltd.	Kantharawichai, Maha Sarakham, Thailand
Dee Khuk Co., Ltd.	Mueang Nakhon Ratchasima, Nakhon Ratchasima, Thailand
Nit Foods Limited Partnership	Sawankhalok, Sukhothai, Thailand
Plara Chef Praitoon Food Products Co., Ltd.	Ubolratana, Khon Kaen, Thailand
Thepthida Food Co., Ltd	Prachaksinlapakhom, Udon Thani, Thailand
1999 Khon Kaen Food Products	Mueang Khon Kaen, Khon Kaen, Thailand
